# Attitudes towards suicide attempts broadcast on social media: an exploratory study of Chinese microblogs

**DOI:** 10.7717/peerj.1209

**Published:** 2015-09-08

**Authors:** Ang Li, Xiaoxiao Huang, Bibo Hao, Bridianne O’Dea, Helen Christensen, Tingshao Zhu

**Affiliations:** 1 Department of Psychology, Beijing Forestry University, Beijing, China; 2 Black Dog Institute, University of New South Wales, Sydney, NSW, Australia; 3 Institute of Psychology, Chinese Academy of Sciences, Beijing, China; 4 School of Computer and Control, University of Chinese Academy of Sciences, Beijing, China; 5 Institute of Computing Technology, Chinese Academy of Sciences, Beijing, China

**Keywords:** China, Social media, Stigma, Microblog, Suicide, Weibo

## Abstract

**Introduction.** Broadcasting a suicide attempt on social media has become a public health concern in many countries, particularly in China. In these cases, social media users are likely to be the first to witness the suicide attempt, and their attitudes may determine their likelihood of joining rescue efforts. This paper examines Chinese social media (Weibo) users’ attitudes towards suicide attempts broadcast on Weibo.

**Methods.** A total of 4,969 Weibo posts were selected from a customised Weibo User Pool which consisted of 1.06 million active users. The selected posts were then independently coded by two researchers using a coding framework that assessed: (a) Themes, (b) General attitudes, (c) Stigmatising attitudes, (d) Perceived motivations, and (e) Desired responses.

**Results and Discussion.** More than one third of Weibo posts were coded as “stigmatising” (35%). Among these, 22%, 16%, and 15% of posts were coded as “deceitful,” “pathetic,” and “stupid,” respectively. Among the posts which reflected different types of perceived motivations, 57% of posts were coded as “seeking attention.” Among the posts which reflected desired responses, 37% were “not saving” and 28% were “encouraging suicide.” Furthermore, among the posts with negative desired responses (i.e., “not saving” and “encouraging suicide”), 57% and 17% of them were related to different types of stigmatising attitudes and perceived motivations, respectively. Specifically, 29% and 26% of posts reflecting both stigmatising attitudes and negative desired responses were coded as “deceitful” and “pathetic,” respectively, while 66% of posts reflecting both perceived motivations, and negative desired responses were coded as “seeking attention.” Very few posts “promoted literacy” (2%) or “provided resources” (8%). Gender differences existed in multiple categories.

**Conclusions.** This paper confirms the need for stigma reduction campaigns for Chinese social media users to improve their attitudes towards those who broadcast their suicide attempts on social media. Results of this study support the need for improved public health programs in China and may be insightful for other countries and other social media platforms.

## Introduction

Suicide is a leading cause of death worldwide. According to World Health Organization, from 2000 to 2012, over 800,000 people in the world and 7.8 per 100,000 people in China died by suicide each year. It is estimated that over 90% of those who died by suicide also experienced a mental illness or extreme psychological distress (Mann, 2002; Nock et al., 2008). A previous suicide attempt is a leading predictor of suicide. In United States, one suicide occurs for every 25 attempts (Crosby et al., 2011); while, in China, one suicide occurs for every seven attempts (Centers for Disease Control and Prevention, 2004). Longitudinal studies on individuals who had their suicide attempts interrupted found that more than 90% did not re-attempt (Owens, Horrocks & House, 2002). This suggests that efficient intervention during an attempt is critical to preventing death. Because many suicide attempts are sudden, it is a challenge to intervene in a timely manner. Many individuals do not seek help for their suicidal thoughts because of the stigma associated with mental illness (Calear, Batterham & Christensen, 2014). Suicide attempts may be especially stigmatised, dismissed as “merely attention-seeking gesturers” (Sudak, Maxim & Carpenter, 2008). Stigma can perpetuate a mental illness, decreasing quality of life (Link et al., 2001; El-Badri & Mellsop, 2007). A reduction in the stigma of suicide is considered to be a key step in reducing of its toll (Niederkrotenthaler et al., 2014).

In recent years, social media has become an increasingly popular way for people to exchange information in real-time. In China, the most popular Chinese microblogging service provider, Sina Weibo (weibo.com), has over 500 million registered users, producing more than 100 million microblogs per day. Similar to Twitter, Weibo is a free social media site that enables registered users to communicate with others in real-time using posts, which are limited to 140 Chinese characters in length. Weibo users create a network by following other Weibo accounts. The large majority of Weibo content is publically available for viewing and downloading, although some users opt to privatise their accounts. On social media, users are motivated to disclose frequently (Java et al., 2007), and some individuals have used these platforms to broadcast their suicide attempts (Murano, 2014). According to our summary of relevant news stories, in China alone at least 51 cases have been reported since August 2010, with more than half of these cases (27) referring to Weibo. To date, there has only been one study examining the online attitudes towards those who broadcast their suicide attempts on Weibo. In this study, Fu et al. (2013) found that 23% of the responses to the broadcast could be described as cynical or indifferent. This conflicts with responses to the suicide death of Robin Williams, for which the overwhelming emotional response on Twitter was one of sadness (Larsen et al., 2015). As those in the online social network are likely to be the first to witness a suicide attempt, their responses may indicate their willingness to join in rescue efforts or instead, hamper recovery by being negative and unhelpful (Niederkrotenthaler et al., 2014; Haimson et al., 2014). Understanding the ways in which suicide stigma presents online is crucial to better understanding whether the online social network can be harnessed to intervene and prevent death (Boudewyns et al., 2015).

This study aims to examine Weibo users’ attitudes towards those who broadcast their suicide attempts online by analysing Weibo posts related to this topic. Based on Fu et al.’s (2013) research, it is hypothesised that a meaningful portion (∼20%) of the posts will be stigmatising.

## Methods

This study consisted of two steps: (i) Data collection; and (ii) Coding. Methods and procedures of this study were approved by the Institutional Review Board of the Institute of Psychology, Chinese Academy of Sciences. Informed consent was not obtained from the Weibo users as the data used was publicly available.

### Data collection

**Figure 1:**
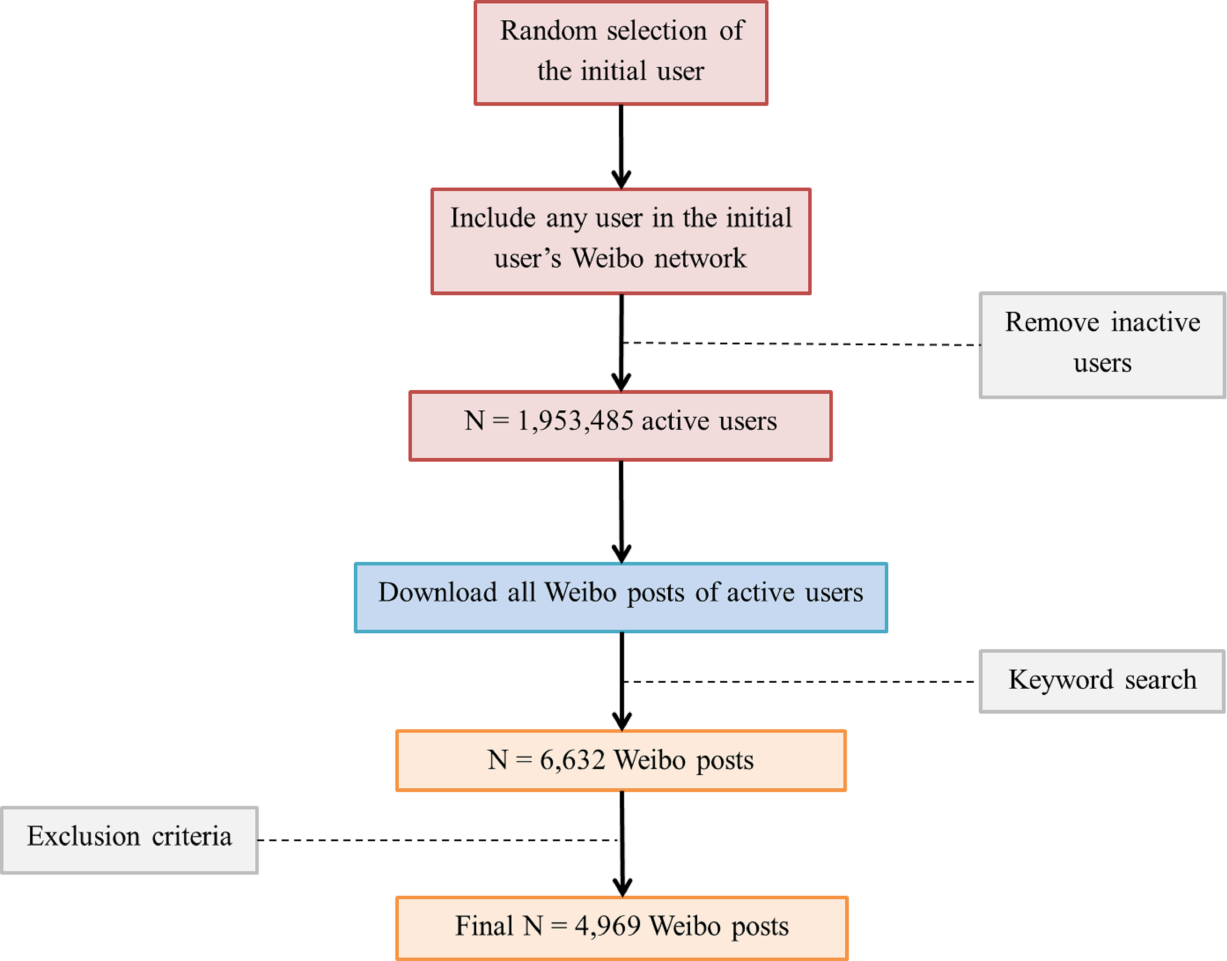
Process of data collection.

Data was collected using a three-step procedure: (1) Selection of Weibo users; (2) Collection of Weibo posts; and (3) Filtering out irrelevant posts (see Fig. 1).

#### Selection of Weibo users

**Figure 2:**
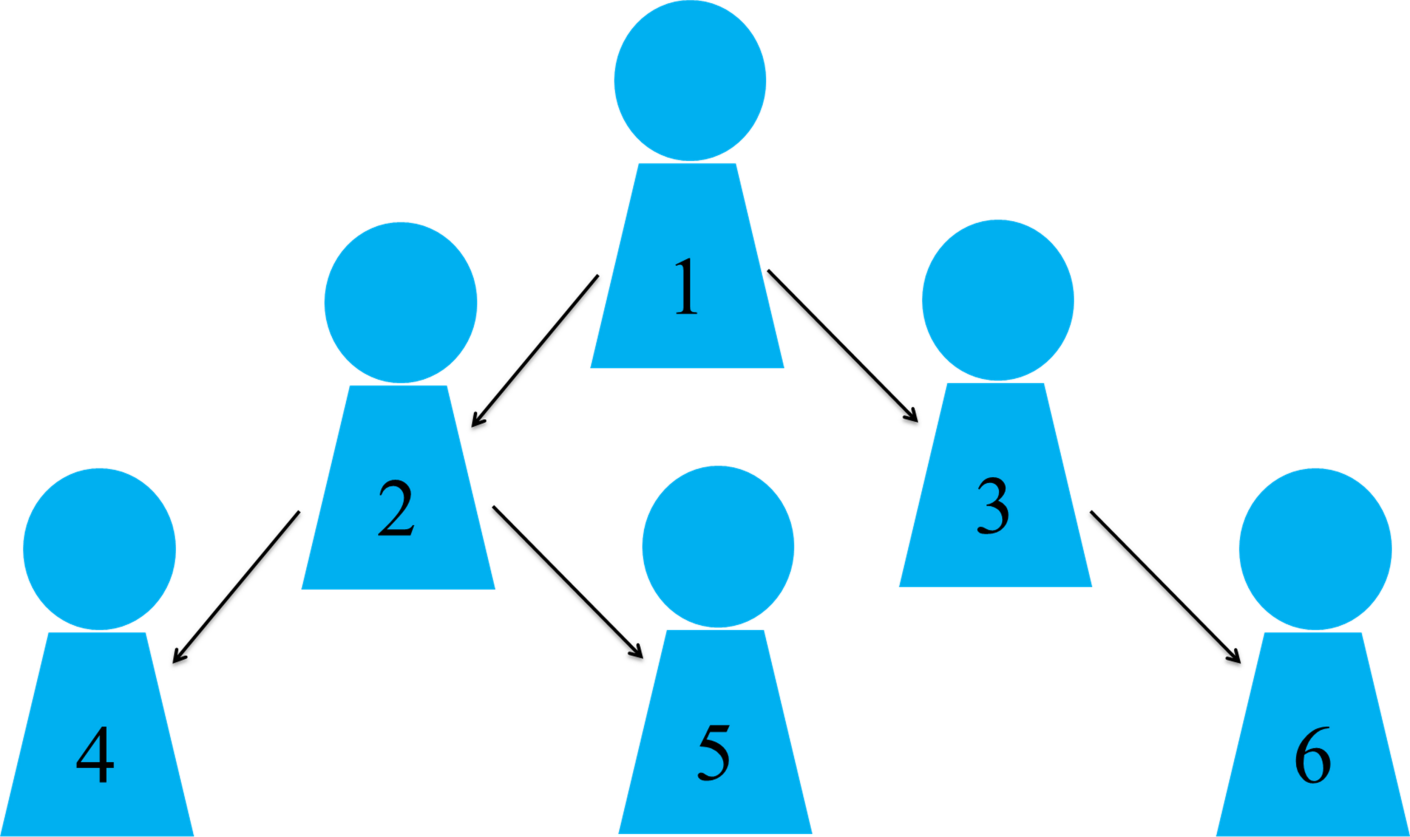
Order in which Weibo users are expanded to be included in the WUP.

To build the Weibo User Pool (WUP), a breadth-first search algorithm for searching tree data structures, was utilised (Skiena, 2008). Specifically, the search starts at the tree root (an initial user) and explores the neighbour nodes first (online friends of the initial user), before moving to the next level neighbours (online friends of the initial user’s friends) (see Fig. 2). Beginning in 2012, one initial Weibo user (the seed user) was randomly selected. The pool was then expanded to include any user in the seed user’s Weibo network. This study aimed to discern “active” Weibo users who are likely to be more engaged with intervention efforts. Users were determined to be “active” based on their total number of posts, and average number of daily posts. In previous research on Weibo (Li et al., 2014), the distribution of all individual users’ total number of posts (136.65 ± 788.87) and average number of daily posts (2.84 ± 2.57) were examined and used to calculate the necessary values. In the current study, active users were defined as those with: (a) at least 532 published Weibo posts (136.65 + 0.5 × 788.87 ≈ 532); (b) an average number of daily posts ranging from 2.84 (the mean value of all individual users’ average number of daily posts) to 40 (a threshold for excluding extreme users, like movie or sports stars, who might update Weibo posts for business purpose). Any user who did not post in the three months prior to data collection or whose latest posts were updated within one month of registration was excluded.

#### Collection of Weibo posts

In compliance with Weibo’s privacy and data access control policy, Weibo posts were collected using the Application Programming Interfaces (API), which allowed access to the Weibo posts of any specific user. Gender and location of the Weibo users are listed in their registration information and can also be downloaded through API. All Weibo posts of active users were downloaded to a database, which also automatically downloaded any new posts. Changes in user accounts and data accessibility in the final data set (e.g., data downloads were prohibited by service provider due to the data access control policy, and user accounts or Weibo posts were removed by either users themselves or service provider) impacted on collection of posts. Final data collection was undertaken on 9th March 2015.

#### Filtering out irrelevant posts

To identify the posts that were related to an online suicide, the posts were searched using a set of specific keywords which included: “live broadcast of suicide” (自杀直播, zisha 直播, suicide 直播, 直播自杀, 直播 zisha, 直播 suicide); “self-presentation of suicide” (秀自杀, 秀 zisha, 秀 suicide, 晒自杀, 晒 zisha, 晒 suicide); and “suicide show” (自杀秀, zisha 秀, suicide 秀). A total of 6,632 Weibo posts with one or more of the keywords were identified. Further scrutiny of the posts occurred to exclude the following: (a) posts which focused on any topic other than the broadcasting of a suicide attempt on social media (e.g., movie/TV programs, or broadcast of a suicide attempt in the real world) or posts which used relevant keywords for non-suicidal purposes (e.g., making a promise, or making a bet); (b) posts which reflected one’s own suicide attempt other than one’s attitudes towards those who broadcast their suicide attempts on social media; (c) posts which discussed any attempter whose suicidal posts were released after death (e.g., the attempter known as Zoufan (走饭)); (d) posts published in a language other than Chinese or English; (e) posts which could not be coded accurately due to lack of context or the use of keywords only.

### Coding

A content analysis of the posts was conducted using Microsoft Excel spreadsheet. The coding framework was developed by consensus and is based on previous research investigating the stigma of suicide (Batterham, Calear & Christensen, 2013a; Batterham, Calear & Christensen, 2013b). To generate a coding framework, one researcher analysed collected Weibo posts inductively, and then classified and regrouped them into an initial framework. Then, another two researchers were recruited to conduct the classification. After a training session, the two researchers provided feedback. The initial framework was amended accordingly and the final coding framework was developed. Using the framework outlined in Table 1, two researchers were instructed to classify the theme, attitude, perceived motivation, and desired response for each post using the subcategories provided. For posts with an attitude categorised as “stigmatising,” coders were required to further classify this attitude as being “stupid, shallow, embarrassing, deceitful, vengeful, weak, selfish, immoral, pathetic, glorified/normalised, or strange.” If a post contained content that did not fit with the primary categories, the post was coded as “irrelevant” for that category. All posts were coded independently. Discrepancies between two coders were resolved by a third researcher’s decision.

### Statistical analysis

Descriptive analyses were conducted to provide gender and location information of the dataset, as well as the results of the coding task. Cohen’s *κ* coefficients were calculated to measure the agreement between two coders. To examine gender differences, a series of *χ*^2^ tests using an alpha level of 0.05 were conducted.

## Results

### Participants

A total of 99,925,821 Weibo users were included in the final WUP. Of these, 1,953,485 were identified as being active users. Of these active users, 1.06 million were able to have posts extracted using the API. A total of 6,632 posts from these active users matched the keywords. Of these posts, a further 1,663 were excluded. The final sample consisted of 4,969 posts from 4,582 distinct Weibo users. The gender and location of the users are shown in Table 2.

### Coding

Table 3 presents the results of the coding task. The Cohen’s *κ* coefficients for themes, general attitudes, stigmatising attitudes, perceived motivations, and desired responses for the human coders were 0.82, 0.85, 0.73, 0.73, and 0.67, respectively.

#### Themes

All of the 4,969 Weibo posts (male: 2,721 posts; female: 2,183 posts) were related to different types of themes. Gender differences in the four types of themes were significant. Males were more likely than females to have posts coded as “distributing news” (*χ*^2^ = 104.37, *df* = 1, *p* < 0.001) and “providing resources” (*χ*^2^ = 6.92, *df* = 1, *p* < 0.01); whereas females were more likely to have posts coded as “sharing experience” (*χ*^2^ = 31.30, *df* = 1, *p* < 0.001) and “discussing case” (*χ*^2^ = 33.52, *df* = 1, *p* < 0.001). No other significant gender differences were found.

#### General attitudes

All of the 4,969 Weibo posts were related to different types of general attitudes. Males were more likely than females to have posts coded as “neutral” (*χ*^2^ = 81.36, *df* = 1, *p* < 0.001); whereas females were more likely to have posts coded as “stigmatising” (*χ*^2^ = 42.48, *df* = 1, *p* < 0.001). No other significant gender differences were found.

#### Stigmatising attitudes

A total of 1,760/4,969 Weibo posts were coded as indicating a stigmatising attitude (male: 858 posts; female: 884 posts). Males were more likely than females to have posts coded as “immoral” (*χ*^2^ = 9.68, *df* = 1, *p* < 0.01) and “glorified/normalised” (*χ*^2^ = 3.94, *df* = 1, *p* < 0.05). No other significant gender differences were found.

#### Perceived motivations

A total of 480/4,969 Weibo posts were coded as referencing a perceived motivation (male: 234 posts; female: 238 posts). Females were more likely than males to have posts coded as “threatening” (*χ*^2^ = 4.58, *df* = 1, *p* < 0.05). No other significant gender differences were found.

#### Desired responses

A total of 394/4,969 Weibo posts were coded as reflecting a particular type of desired response (male: 226 posts; female: 164 posts). No significant gender differences were found.

Among the posts reflecting a negative desired response (i.e., “not saving” and “encouraging suicide”; *n* = 257), 57% of these were also stigmatising (all: 147; male: 81; female: 65) and 17% also commented on the perceived motivations (all: 44; male: 19; female: 25). Among those who had a negative response and stigmatising post (*n* = 147), males were more likely to consider the attempt “selfish” (*χ*^2^ = 5.90, *df* = 1, *p* < 0.05) and to “glorify/normalise” the attempt (*χ*^2^ = 5.02, *df* = 1, *p* < 0.05); whereas females were more likely to consider the attempt “deceitful” (*χ*^2^ = 4.58, *df* = 1, *p* < 0.05) and “vengeful” (*χ*^2^ = 9.16, *df* = 1, *p* < 0.01). No other significant gender differences were found.

## Discussion

This study used human coders to analyse 4,969 Weibo posts to explore users’ attitudes towards those who broadcast their suicide attempts online. The range of Cohen’s *κ* coefficients suggested a substantial and acceptable level of agreement (Landis & Koch, 1977). This study confirmed that Weibo is used as a platform to share thoughts and beliefs about the topic of online suicides. Suicide stigma was evident. More than one third of the posts were found to be “stigmatising” and females were more likely to be stigmatising than males. Terms such as “deceitful,” “pathetic,” and “stupid” were commonly used to describe the attempters. Males were more likely than females to believe that an attempt was “immoral” and worthy of “glorification.” Consistent with previous research (Sudak, Maxim & Carpenter, 2008), “attention seeking” was considered to be the primary motivator for the attempt and females were more likely to believe attempts were motivated by a desire to “threaten others” or “gain an advantage.” Many of the posts coded as having a “not saving” and “encouraging suicide” response were also found to include a stigmatising attitude, indicating that stigmatising beliefs may result in an unwillingness to seek help for the attempter. Sadly, no more than 10% of the posts included references to mental health literacy or mental health information. Overall, the large majority of Weibo users’ attitudes to suicide appeared to be discordant with the truth about suicide, indicating a limited knowledge of the complexity of suicidality including its causes, signs and symptoms, risk factors, and effective treatment and prevention (Jorm, 2000; Batterham, Calear & Christensen, 2013a). This is consistent with other research on suicide attitudes in China and other Asian countries (Khan, 2002; Tzeng & Lipson, 2004; Lee et al., 2007).

This study has a number of implications. First, attempts to reduce suicide stigma must continue. Second, such attempts should be gender-specific (Griffiths, Christensen & Jorm, 2008; Batterham et al., 2013). Improving mental health literacy is an effective strategy to improve stigmatising attitudes and reduce misleading beliefs (Paykel, Hart & Priest, 1998; Daigle et al., 2006; Sun, Long & Boore, 2007; Tzeng et al., 2010). Social media campaigns allow for accelerated exposure and network penetration (Betton et al., 2015), although effective stigma reduction is yet to be replicated online. Whilst social media campaigns have been found to improve attitudes towards mental health issues, they appear to be less effective at providing the tools people need to feel capable of helping someone who may be experiencing a mental health issue (Livingston et al., 2014). This is crucial for suicide prevention efforts. Whilst normalising suicidal behaviours is not the goal, this study emphasises the need for revised campaigns to challenge negative attitudes towards suicide and mental illness whilst also promoting help-seeking. In particular, stigma campaigns need to address beliefs about “attention seeking” and character judgements such as “deceitful” and “pathetic” whilst taking into account the gender differences. In the event of an online suicide attempt, programs such as “Stigma Watch” may be helpful (https://www.sane.org/stigmawatch), but more evaluative research is needed to determine best practice for reducing suicide in the event of an online attempt.

It is important to note the limitations of this study. The findings may have limited generalisability. First of all, attitudes to social media suicide may not be the same as attitudes to offline suicide. Secondly, social media users form only a sample of the Chinese population: 63% of Chinese social media users are aged between 10 and 29 years (China Internet Network Information Center, 2012), suggesting that social media users are not representative of all people in China. The analysis of the current study focused on public Weibo posts. As such, private data may lead to different discoveries, and different results may be found on other social media platforms. Given that a number of online suicide broadcasts occurred outside of Weibo, further investigation of this phenomenon in other online domains is warranted. Finally, it cannot be guaranteed that users in this sample had not attempted suicide in this way. Future work would benefit from clarifying the sample to better understand stigma. Despite these shortcomings, the process of collecting and analysing Weibo posts was conducted in a non-intrusive manner under non-experimental conditions such that Weibo users were not aware that their posts would be analysed in this way. Therefore, the current study has high ecological validity and is likely to reflect the participants’ actual attitudes towards those who broadcast their suicide attempts online.

## Conclusions

Stigma is prominent among Weibo users’ responses to suicides broadcast on social media. Whilst some posts were found to be supportive, the majority indicated negative and unhelpful sentiment. The findings of this study indicate that suicide prevention efforts in these circumstances may be significantly challenged by the level of stigma among the online social network. Sincere, evidence-based efforts to reduce stigma and improve attitudes among Weibo users is needed. This study gives insight into the types of messages that are needed and confirms the importance of gender-specific messaging. In general, messages about literacy promotion and resources are needed to reduce the belief that people broadcasting their attempts on social media are deceitful, pathetic, and attention seeking. Social media platforms are increasingly popular, and present a viable platform for stigma reduction campaigns, although research is in its infancy. This study supports further investigation of the ways in which suicide stigma spreads and changes online, to better inform reduction campaigns and public health initiatives. The detailed analysis of the Weibo posts provides a useful framework for further analysis of suicide stigma in China and other countries, and may be adapted to other stigmatised health problems.

**Table 1:** Outline of coding framework.

Primary category	Definition	Example Weibo post
**Themes**		
Sharing experience	Sharing personal experience as a suicide attempter or a witness	“I just saw a person broadcasting suicide live on Weibo…”
Discussing case	Discussing details about a specific case (e.g., personal information, suicide motivation, suicide method, situation and progress, and the final consequence)	“Whether or not that person broadcasting suicide live on Weibo has died??”
Distributing news	Describing or linking relevant news	“A man broadcasting live suicide has been saved by other users http://...”
Promoting literacy	Providing professional information to improve mental health literacy or raise public awareness, and encouraging discussion about suicide attempts broadcast on social media	“I really have followed through with the whole process! Really died! The nature of broadcasting live suicide needs to be examined from the perspective of psychology or communication.”
Providing resources	Providing advice and support to attempters or calling for help	“It seems to be live broadcast of suicide…Help.”
Expressing general opinion	Expressing one’s general opinion towards suicide attempts broadcast on social media	“Live broadcast of suicide has become such a recent popular trend…”
**General attitudes**		
Supportive	Sympathising and encouraging the individual making the attempt	“It is my first time seeing live Weibo broadcast of suicide. I have been feeling really sad the whole night…sigh.”
Neutral	Indicating a neutral attitude towards the suicide attempt	“Weibo has become a popular place for broadcasting live suicide”
Unpleasant	Having a feeling of discomfort, unhappiness, and revulsion towards the suicide attempt	“Someone is broadcasting live suicide? It is horrible.”
Stigmatising	Disgracing and dishonouring the suicide attempt	“All the people who commit suicide are stupid. However, the people who broadcast suicide live on Weibo are the most stupid among all 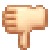 ”
**Stigmatising attitudes**		
Stupid	Belief that those broadcasting their suicide attempts are silly or unwise	“Broadcasting suicide live on Weibo is the stupidest, stupidest, and stupidest act in the world!”
Shallow	Belief that those broadcasting their suicide attempts show a lack of serious or careful thought	“#A Sichuan girl broadcasted live suicide# It means the opposite of the idiom ‘thinking twice before acting’. She is such a reckless person.”
Embarrassing	Belief that the suicide attempt is shameful	“Live broadcast of suicide. Why. It is a bad influence. Committing suicide is too private to share. BBQ is much better than charcoal-burning suicide.”
Deceitful	Belief that the suicide attempt is fake	“Don’t be fooled by them, how can we ensure that people broadcasting live suicide really want to die??”
Vengeful	Belief that those broadcasting their attempts express a strong wish to punish someone	“The man broadcasting live suicide did not appreciate the life he had. If so, how can he be expected to appreciate some else’s life? If he was still alive, who knows whether he would launch a suicide bombing attack on a bus 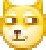 ”
Weak	Belief that those broadcasting their attempts show a lack of strength and cannot sustain pressure	“How could the person be so weak? Live Weibo broadcast of suicide ” 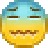
Selfish	Belief that those broadcasting their attempts only think of their own advantage	“I saw a person broadcasting suicide live on Weibo just before sleep 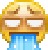 . That man doesn’t love you anymore. Killing yourself is never worth it! You are such a selfish person! Taking your time and thinking about your parents!”
Immoral	Belief that the attempt violates some moral laws, norms or standards	“#A Sichuan girl broadcasted live suicide# Live broadcast of suicide sets a very bad example for children”
Pathetic	Belief that those broadcasting their attempts do not deserve to be respected, due to their unsuccessfulness and uselessness	“When I came back at night, that boy broadcasting suicide live on Weibo in the morning has died. Thanks for Darwin’s theory of evolution by natural selection.”
Glorified/normalised	Belief that the attempt is a personal right or a sign of noble souls, bravery and strength	“Live Weibo broadcast of suicide. So cool! http://...”
Strange	Belief that it is difficult to understand those who broadcast their attempts	“I can understand why people commit suicide. But I can never understand why people commit such an unusual live broadcast of suicide.”
**Perceived motivations**		
Seeking attention	The reason for broadcasting the attempt is to make oneself famous on social media	“Having many followers is so important. If not, few persons can introduce your live broadcast of suicide to others. What a shame.”
Seeking help/support	The reason for broadcasting the attempt is to seek help or support from others	“ ‘Live broadcast of suicide’ is an act of ‘performing help-seeking behaviour’ http://...”
Threatening	The reason for broadcasting the attempt is to threaten others to gain an advantage	“The man broadcasting live suicide doesn’t really want to die. He probably wants to get his ex-girlfriend back. The person who really wants to die should only leave a suicide note and kill oneself quietly.”
Escaping	The reason for broadcasting the attempt is to escape day-to-day problems (e.g., stress, family arguments, difficulties at work, and financial difficulties)	“A couple of hours ago, one person broadcasted live suicide and another person engaged in live elopement. Although both of them are motivated to escape from reality, they have gone to separate places now. Bless them all!”
Suffering from mental illness	The reason for broadcasting the attempt is due to mental illness	“#A Sichuan girl broadcasted live suicide# I have read her posts. I think she is mentally ill…Be quick to receive treatment at any hospital. I have already recovered from my mental illness 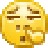 ”
**Desired responses**		
Saving and supporting	Indicating great willingness to save and support those who broadcast their attempts	“I just read a message saying a DJ, who works at a broadcast station, broadcasted suicide live on Weibo. He has 70 thousand followers on Weibo, but there was not even one person who did any help for him at that time…. When you meet people who go through similar situations, please do something to help them. We must help them. Thanks all.”
Saving but separating	Indicating willingness to save those who broadcast their attempts, but advocating to separate them from others	“It is my response to people broadcasting suicide live on Weibo. No reports, no comments, no forwards, but calling the police! Because any comment or forward might increase pressure on people with depression and inspire others to put their suicidal ideas into practice. My response should be good for both individuals and communities.”
Saving but punishing	Indicating willingness to save those who broadcast their attempts, but advocating to criticise, shame, and punish them	“To people broadcasting suicide live on Weibo, we should give them a good scolding after saving them!”
Not saving	Indicating reluctance to save those who broadcast their attempts	“Do not save people broadcasting live suicide! You have the right to kill yourself, but I don’t have a responsibility to save your life.”
Encouraging suicide	Indicating an intention to encourage people, who broadcast their attempts, to complete suicide	“Please be quick! Anyone who broadcasts suicide live on Weibo doesn’t deserve to be respected! 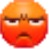 ”

**Table 2:** Demographics of participants (*N* = 4,582).

Category	*n*	%
**Gender**		
Male	2,493	54
Female	2,040	45
Not specified	49	1
**Location**		
Anhui	60	1
Aomen	3	0.10
Beijing	551	12
Chongqing	83	2
Fujian	115	3
Gansu	10	0.20
Guangdong	811	18
Guangxi	73	2
Guizhou	35	1
Hainan	25	1
Hebei	52	1
Henan	73	2
Heilongjiang	41	1
Hong Kong	21	1
Hubei	93	2
Hunan	51	1
Inner Mongolia	26	1
Jilin	34	1
Jiangsu	240	5
Jiangxi	46	1
Liaoning	99	2
Ningxia	6	0.10
Qinghai	2	0.04
Shandong	100	2
Shanxi	28	1
Shaanxi	79	2
Shanghai	646	14
Sichuan	227	5
Taiwan	4	0.10
Tianjin	54	1
Tibet	2	0.04
Xinjiang	19	0.40
Yunnan	59	1
Zhejiang	237	5
International	259	6
Not specified	318	7

**Table 3:** Coding results.

		%	(*n*)
**Themes**			**N** = **4,969**
	Discussing case	49	(2,439)
	Distributing news	18	(889)
	Expressing general opinion	14	(698)
	Sharing experience	9	(440)
	Providing resources	8	(405)
	Promoting literacy	2	(98)
**General attitudes**			**N** = **4,969**
	Neutral	26	(1,299)
	Supportive	23	(1,132)
	Unpleasant	16	(778)
	Stigmatising	35	(1,760)
	*Deceitful*	*22*	(388)
	*Pathetic*	*16*	(287)
	*Stupid*	*15*	(268)
	*Shallow*	*9*	(166)
	*Immoral*	*9*	(151)
	*Embarrassing*	*8*	(142)
	*Selfish*	*6*	(109)
	*Strange*	*6*	(101)
	*Weak*	*4*	(63)
	*Vengeful*	*3*	(57)
	*Glorified/normalised*	*2*	(28)
**Perceived motivations**			**N** = **480**
	Seeking attention	57	(275)
	Threatening	13	(60)
	Seeking help/support	12	(59)
	Escaping	10	(48)
	Suffering from mental illness	8	(38)
**Desired responses**			**N** = **394**
	Negative–not saving	37	(146)
	Negative–encouraging suicide	28	(111)
	Saving and supporting	18	(72)
	Saving but separating	10	(38)
	Saving but punishing	7	(27)
**Negative desired responses AND stigmatising**			**N** = **147**
	Deceitful	29	(43)
	Pathetic	26	(38)
	Embarrassing	12	(17)
	Immoral	10	(14)
	Shallow	5	(7)
	Vengeful	5	(7)
	Selfish	5	(7)
	Glorified/normalised	4	(6)
	Stupid	3	(4)
	Strange	2	(3)
	Weak	1	(1)
**Negative desired responses AND perceived motivations**			**N** = **44**
	Seeking attention	66	(29)
	Threatening	16	(7)
	Seeking help/support	9	(4)
	Escaping	5	(2)
	Suffering from mental illness	5	(2)

## Supplemental Information

10.7717/peerj.1209/supp-1 Supplemental Information 1 Data
